# Effect of heterologous expression of *FT* gene from *Medicago truncatula* in growth and flowering behavior of olive plants

**DOI:** 10.3389/fpls.2024.1323087

**Published:** 2024-02-22

**Authors:** Consuelo Guerrero, Sergio Cerezo, Isabel Feito, Lucía Rodríguez, Alon Samach, José A. Mercado, Fernando Pliego-Alfaro, Elena Palomo-Ríos

**Affiliations:** ^1^ Departamento de Botánica y Fisiología Vegetal, Instituto de Hortofruticultura Subtropical y Mediterránea ‘La Mayora’, Universidad de Málaga, Spanish National Research Council (IHSM-UMA-CSIC), Málaga, Spain; ^2^ Servicio Regional de Investigación y Desarrollo Agroalimentario de Asturias, Finca Experimental “La Mata”, Grado, Spain; ^3^ The Robert H. Smith Institute of Plant Sciences and Genetics in Agriculture, The Robert H. Smith Faculty of Agriculture, Food and Environment, The Hebrew University of Jerusalem, Rehovot, Israel

**Keywords:** *Olea europaea* L. subsp. *europaea*, FT gene, TFL gene, flowering, branching phenotype, hormonal content

## Abstract

Olive (*Olea europaea* L. subsp. *europaea*) is one of the most important crops of the Mediterranean Basin and temperate areas worldwide. Obtaining new olive varieties adapted to climatic changing conditions and to modern agricultural practices, as well as other traits such as biotic and abiotic stress resistance and increased oil quality, is currently required; however, the long juvenile phase, as in most woody plants, is the bottleneck in olive breeding programs. Overexpression of genes encoding the ‘florigen’ Flowering Locus T (FT), can cause the loss of the juvenile phase in many perennials including olives. In this investigation, further characterization of three transgenic olive lines containing an FT encoding gene from *Medicago truncatula, MtFTa1*, under the 35S CaMV promoter, was carried out. While all three lines flowered under *in vitro* conditions, one of the lines stopped flowering after acclimatisation. In soil, all three lines exhibited a modified plant architecture; e.g., a continuous branching behaviour and a dwarfing growth habit. Gene expression and hormone content in shoot tips, containing the meristems from which this phenotype emerged, were examined. Higher levels of *OeTFL1*, a gene encoding the flowering repressor TERMINAL FLOWER 1, correlated with lack of flowering. The branching phenotype correlated with higher content of salicylic acid, indole-3-acetic acid and isopentenyl adenosine, and lower content of abscisic acid. The results obtained confirm that heterologous expression of *MtFTa1* in olive induced continuous flowering independently of environmental factors, but also modified plant architecture. These phenotypical changes could be related to the altered hormonal content in transgenic plants.

## 1 Introduction

Olive (*Olea europaea* L. subsp. *europaea* var. *europaea*) is one of the oldest crops in the Mediterranean Basin, its origin dates back to the Middle East around 6,000 years ago. It was probably domesticated from its wild progenitor, the oleaster (*Olea europaea* L. subsp. *europaea* var. *sylvestris*), in the area between Turkey and Syria (Zohary and Spiegel-Roy, 1975; Baldoni et al., 2006; Besnard et al., 2013; Diez et al., 2015). Olive oil consumption is steadily increasing worldwide because of its nutritional, therapeutic and organoleptic properties. Its well-balanced fatty acid composition, with a higher proportion of oleic acid (Contreras et al., 2020), contributes to reducing the risk of cardiovascular diseases, moreover, the presence of antioxidant compounds such as phenolic compounds (e.g. oleuropein), vitamin E and w-3 fatty acids (α–linolenic acid), makes olive oil a valuable product to introduce in the diet (Conde et al., 2008; Kiritsakis and Shadini, 2017). Today, olive remains the most economically important oil tree crop in temperate areas worldwide, with 12.7 million ha under cultivation (FAOSTAT, 2020).

Olive cultivars and their wild relatives (oleasters) are diploid (2n = 2x = 46), predominantly allogamous, and interfertile (Belaj et al., 2007). Wild olives reproduce sexually by wind pollination, while olive cultivars are currently propagated by cuttings, grafting (Baldoni et al., 2006; Alagna et al., 2019) and nodal segments *in vitro* (Lambardi et al., 2013). One of the most striking difficulties in olive breeding, as in many woody plants (Zhang et al., 2010; Srinivasan et al., 2012; Klocko et al., 2016), lies in its long juvenile phase, which can last up to 15-20 years. This fact has hindered the development of new varieties (Diez et al., 2015; Rallo et al., 2018). Biotechnological approaches to improve this crop are also difficult to apply due to the recalcitrance of olive tissues to regenerate *in vitro*. Along this line, few transgenic olive plants have been generated (Palomo-Ríos et al., 2021).

The transition from the vegetative to the reproductive stage, in annuals as well as in woody perennial plants, appears to be determined by the balance between the floral integrator FLOWERING LOCUS T (FT) and the flowering repressor TERMINAL FLOWER 1 (TFL1), both homologues of the phosphatidylethanolamine-binding protein (PEBP) family (Hanzawa et al., 2005; Ahn et al., 2006; Laurie et al., 2011; Brunner et al., 2014; Putterill and Varkonyi-Gasic, 2016). *FT* and *TFL1* genes encode related proteins with opposite functions: FT induces flowering while TFL1 represses it. In *Arabidopsis thaliana*, the swap of a single amino acid is enough to convert TFL1 into FT function and vice versa (Hanzawa et al., 2005; Ahn et al., 2006; Ho and Weigel, 2014); in this species, flowering occurs under long days when the floral promoter CONSTANS (CO) activates the expression of the *FT* gene (Samach et al., 2000), producing a small mobile protein (FT, the major florigen) in the companion cells of the phloem in the leaf veins (Amasino and Michaels, 2010; Yoo et al., 2013; Jin et al., 2015; Chen et al., 2018). Later, FT enters the sieve elements and is translocated via the phloem to the shoot apical meristem (Corbesier et al., 2007), where it recruits the bZIP transcription factor FD (Wigge et al., 2005), forming a complex, together with 14-3-3 protein (Taoka et al., 2011, Taoka et al., 2013), that initiates flowering by activating the expression of floral meristem-identity MADS BOX genes such as *APETALA1* (*AP1*), *FRUITFUL* (FUL) *and SUPPRESSOR OF OVEREXPRESSION OF CONSTANS1* (*SOC1*) (Teper-Bamnolker and Samach, 2005; Yoo et al., 2013; Abe et al., 2015). A general model depicting the major events leading to flower induction by long photoperiods in *Arabidopsis* is available (Andres and Coupland, 2012). The effect of cold winter temperatures in promoting FT expression has also been unraveled (Amasino and Michaels, 2010). In other species, the mechanisms that regulate the expression of FT involve transcription factors, biochemical modifications of histones, alternative splicing of mRNA, post-transcriptional control of FT mRNA levels by miRNAs, and post-translational changes of FT (Qin et al., 2017).

The olive tree develops inflorescences from lateral buds in spring after the cold requirements have been fulfilled. Early studies suggested that flower induction in olive occurs before winter, around the time of endocarp sclerification (Rallo and Martin, 1991; Fernandez-Escobar et al., 1992; Rallo et al., 1994; Ulger et al., 2004). Recent evidences indicate that inflorescences are formed at the end of winter, and cold temperatures are likely required for floral induction (Haberman et al., 2017; Engelen et al., 2023). Haberman et al. (2017) identified two *FT* genes in olive, cv. ‘Barnea’, *OeFT1* and *OeFT2*, observing that their expression in mature leaves of adult plants increased at the beginning of winter, from December to February, preceding olive inflorescence initiation. Moreover, the overexpression of *OeFT1* and *OeFT2* in *Arabidopsis thaliana* caused early flowering, showing that these genes could play an essential role in promoting flowering in olive. Artificial application of warm temperatures during winter reduced *OeFT* expression, and olives did not flower (Haberman et al., 2017).

In several perennial species, heavy fruit load (HFL) in one year can inhibit flower induction in the following year, leading to alternate bearing (Samach and Smith, 2013). There is molecular evidence that HFL can increase TFL1 expression in meristems in apples and olives (Haberman et al., 2016, Haberman et al., 2017), as well as reduce *FT* expression in leaves of citrus, mango, avocado and olives (Muñoz-Frambuena et al., 2011; Nakagawa et al., 2012; Ziv et al., 2014; Haberman et al., 2017).

A successful approach aimed at shortening the juvenile phase in perennial woody species has been the overexpression of *FT* genes; e.g., in poplar (Zhang et al., 2010), citrus (Endo et al., 2005), plum (Srinivasan et al., 2012), eucalyptus (Klocko et al., 2016) and olive (Haberman et al., 2017) resulted in early flowering. A different approach that shows promise is silencing of the TFL1 encoding gene (Kotoda et al., 2006; Flachowsky et al., 2012; Freiman et al., 2012), supporting the hypothesis that a coordinated balance between FT and TFL1 levels may be decisive in the transition to flowering (Lifschitz et al., 2014). New strategies in woody plants combining the overexpression of heterologous *FT* genes with suppression of expression of the endogenous *TFL1* homologs using plant viral vectors and VIGS (virus-induced gene silencing) have succeeded in promoting early and continuous flowering in pear and apple (Yamagishi et al., 2014; Yamagishi et al., 2016). In addition to flower induction, the interaction between FT and TFL1 orthologs also controls other processes in plants, such as the indeterminacy of apical meristem and plant architecture (Srinivasan et al., 2012; Brunner et al., 2014; Lifschitz et al., 2014; Klocko et al., 2016; Jin et al., 2021).

The key role of FT genes in olive flowering was demonstrated by the constitutive expression of the *Medicago truncatula MtFTa1* gene in transgenic olive plants (Haberman et al., 2017). Three out of the 9 olive transgenic lines constitutively expressing the *MtFTa1* gene flowered prematurely under *in vitro* conditions. The repetitive conversion of the apical meristems to floral buds caused continuous growth of lateral shoots, an added difficulty in the micropropagation of these lines. After acclimatisation, two of the *in vitro* flowering lines produced new flowers year-round. In the present study, we have characterised the three *MtFTa1* transgenic lines to elucidate the mechanisms underlying flowering in this species. Thus, the effects of *MtFTa1* overexpression on growth habit, hormonal content and endogenous transcript levels of the main olive flowering genes in stem tips were analysed.

## 2 Materials and methods

### 2.1 Plant material

Three transgenic olive lines (FT5, FT7 and FT15) overexpressing the *FT* orthologue gene from *Medicago truncatula* (*Mt*FTa1) were employed in this study. These transgenic lines had been obtained by Haberman et al. (2017) after *Agrobacterium tumefaciens* transformation of the embryogenic line P1, derived from the radicle of a mature zygotic embryo of cv. ‘Picual’ (Torreblanca et al., 2010). Plants from non-transformed control and transgenic lines were regenerated from somatic embryos as described by Cerezo et al. (2011), acclimated to *ex vitro* conditions and maintained for ten years in a confined greenhouse with a cooling system, 30°C maximum temperature, and daylight conditions. Average temperature during the winter season was 16°C, with a minimum temperature of 4.5°C. Plants were grown in 24 cm pots containing a mixture of substrate:sand 70:30 and were fertilized weekly. Three plants per genotype were used.

### 2.2 DNA extraction

Stem tips, including apical and lateral buds and the first two pairs of young developing leaves, were collected from olive plants, washed briefly in distilled water, dried on filter paper, and immediately frozen in liquid nitrogen and stored at -80°C until DNA extraction. Genomic DNA was isolated using the procedure described by Gawel and Jarret (1991). DNA concentration and purity were estimated using a Nanodrop ND-1000 device (Nanodrop Technologies, Inc., Montchanin, DE, United States) and by electrophoresis on agarose gels stained with SYBR Safe (Invitrogen).

### 2.3 Gene copy number analysis of *MtFTa1* by Southern blot

The presence of *MtFTa1* was monitored in the olive transgenic lines by *Southern* analysis. Before digestion, genomic DNA was treated with Ribonuclease A (DNAse-free, Roche), purified by phenol:chloroform:isoamyl alcohol extraction, precipitated using sodium acetate and ethanol and resuspended in sterile deionised water. To determine the transgene copy number, ten µg of DNA were digested sequentially with *Xho*I and *Kpn*I, two restriction enzymes that are not present in the T-DNA of the *35S:MtFTa1* plasmid. Subsequently, DNA was precipitated with sodium acetate and ethanol, air-dried and finally resuspended in 30 µl of sterile deionised water. Then, DNA was fractionated by electrophoresis on 0.8% agarose gel and transferred onto positively-charged nylon membranes using standard procedures, fixed to the filter using UV light and hybridised with the labelled probe. The *35S:MtFTa1* plasmid was used as template for the synthesis of the probe, which was obtained using the “PCR DIG Probe Synthesis Kit” (Roche), 5´-GGAAATCAACCGAGAGTGAG-3´ as forward primer (*MtFTa1*-For), and 5´-AAGAAGACAGCAGCAACAGG-3´ as reverse primer (*MtFTa1*-Rev). Amplification conditions were: 3 min at 95°C, 30 cycles of 45 s, 95°C, 45 s at 55°C, and 1 min 30 s at 72°C, with a final extension step of 7 min at 72°C. PCR was carried out in a final volume of 50 µl in an Eppendorf Mastercycler Personal System, obtaining a PCR product 340 bp long, labelled with digoxigenin-dUTP as a probe.

Hybridisation was performed using the “DIG High Prime DNA Labelling and Detection Starter Kit II” (Roche), according to the manufacturer´s instructions. The membrane was incubated at 42°C for 30 min in the prehybridization solution and then in the hybridisation solution overnight at 42°C. Later, the membrane was washed at room temperature twice for 5 min in (2X SSC, 0.1% SDS) solution and then at 68°C twice for 15 min in (0.5X SSC, 0.1% SDS). The labelled probe was immunodetected with sheep anti-digoxigenin antibody conjugated with alkaline phosphatase and then visualised using the chemiluminescent substrate CSPD (Roche) for alkaline phosphatase.

### 2.4 Total RNA extraction

Stem tips from control and transgenic plants were collected at different times during a growing cycle, spring (April-30-2018), autumn (October-26-2018) and winter (January-22-2019). Once collected, samples were transported on ice, washed in distilled water, dried on filter paper, and immediately frozen in liquid nitrogen and stored at -80°C until RNA isolation. Three independent RNA isolations were carried out per sample, and three technical replicates were used per RNA isolation. Plant material was ground with mortar and pestle using liquid nitrogen and weighted before RNA extraction. Total RNA was isolated using the Spectrum Plant Total RNA Kit (SIGMA), starting with 100 mg of ground plant tissue, according to the manufacturer´s instructions. The concentration and purity of RNA were estimated using a Nanodrop ND-1000 device and by electrophoresis on agarose gels.

### 2.5 cDNA synthesis and gene expression analysis

Before cDNA synthesis, RNA samples were treated with DNase I (RNase-free, Roche), extracted with phenol:chloroform, ethanol-precipitated and quantified in a Nanodrop spectrophotometer. Single-stranded cDNA was synthesised from 1 µg of pure total RNA using iScript cDNA Synthesis Kit (BIO-RAD), according to the manufacturer´s indications. cDNA amplification was evaluated by PCR amplification of the olive ubiquitin gene (Gómez-Jiménez et al., 2010).

The expression of the *MtFTa1* transgene and three endogenous olive genes, *OeFT1*, *OeFT2* and *OeTFL1-1*, during the three sampling periods was monitored by Quantitative real-time PCR (qRT-PCR). qRT-PCR was performed in a final volume reaction of 20 µl containing SsoAdvanced Universal SYBR™ Green Supermix 1X (BIO-RAD), 0.5 µM of each specific primer and 1 µl of diluted cDNA (1:20). Amplification reactions were performed in a CFX96™ Real-Time PCR System (BIO-RAD), using the following PCR program: 95°C for 30 s; 40 cycles at 95°C for 5 s, 60°C for 30 s; and a melting curve from 65°C to 95°C with 0.5°C increments at 5 s intervals. Specific primers used for real-time amplification of each gene are shown in **Supplementary Table 1**. Olive cDNA sequences used for primer design were obtained from the olive cultivar Barnea and the published olive genome data (Cruz et al., 2016; OE5A transcripts databases), and *Medicago truncatula* cDNA used for *MtFTa1* primers were obtained from GenBank, NCBI (Laurie et al., 2011); the accession numbers for the sequences are as follows: *OeFT1* (OE5A107414T1), *OeFT2* (OE5A103537T1), *OeTFL1-1* (OE5A037908T1), and *MtFTa1* (HQ721813.1). Ubiquitin was used as housekeeping gene for normalisation of the expression in all the qRT-PCR experiments (Gómez-Jiménez et al., 2010). Relative amounts of each transcript were calculated with the 2^-ΔΔCT^ method (Livak and Schmittgen, 2001). Three independent RNA isolations were carried out per sample, and three technical replicates were used per RNA isolation. A relative expression value of 1.0 was given to the control with the lowest expression among the three sampling dates evaluated.

### 2.6 Hormonal analyses

The following plant growth regulators, natural and deuterium labelled standards (IDS), were obtained from Sigma Aldrich (St. Louis, MO, USA): abscisic acid, ABA; indol-3-acetic acid, IAA; jasmonic acid, JA; salicylic acid, SA; SA-d6. In addition, benzyladenine, BA; gibberellin, GA_7_; isopentenyl adenosine, iPR; ABA-d6; IAA-d5; BA-d7, were from Olchemin Ltd. (Olomouc, Czech Republic). Accurately weighed solid portions of both natural standards and deuterium-labelled compounds were dissolved in methanol to prepare all the stock working solutions. Methanol (HiperSolv CHROMANOR MS grade) and formic acid were obtained from VWR BDH Chemical (Barcelona, Spain); hydrochloric acid from PanReac (Barcelona, Spain); 2-propanol (super pure solvent) and dichloromethane (HPLC/MS grade) from Romil (Cambridge, UK); ammonium formate from Sigma Aldrich (St. Louis, MO, USA). Deionized Milli-Q water was obtained by means of a Milli-Q® Advantage A10 (Millipore, Darmstadt, Germany).

UHPLC System (1290 Infinity Binary LC System, Agilent Technologies, Madrid, Spain) composed of a binary pump, autosampler, thermostat and DAD detector, was coupled to a 6460 Triple QuadLC/MS fitted with ESI ion source (Agilent Technologies, Madrid, Spain). Data acquisition and processing were performed using MassHunter Workstation software (Agilent Technologies, Madrid, Spain).

Stem tip samples from control and transgenic plants obtained in spring (April-30-2018) were collected, transported in tubes on ice, frozen in liquid nitrogen and stored at -80°C until analysis. The extraction of phytohormones was done from 0.15 g fresh weight according to the protocol of Pan et al. (2010) with modifications from Delatorre et al. (2017). Three biological replicates per line and three technical replicates per sample were used. To enhance matrix removal and obtain better sensitivity and signal-to-noise, four Bond Elut QuEChERS Dispersive Kits were tested. QuEChER 5982-5221 CH (QuEChERS dSPE 2 mL Pigments EN with CH, 100 pk contains 25 mg PSA, 150 mg MgSO4, 2.5 mg GCB; Agilent California, USA) was ultimately selected and used to clean up several samples. Samples were injected into UHPLC System (1290 Infinity binary LC system, Agilent Technologies, Madrid Spain). The UHPLC was coupled to a Triple Quadrupole (6460 Triple Quad, LC/MS equipped with ESI-Ion Source). All compounds were separated and quantified following the protocol described by Delatorre et al. (2017).

### 2.7 Characterisation of flowering behaviour in FT7 plants

The flowering behaviour of FT7 plants, a transgenic line which flowered all year round in the greenhouse, was characterised at three time points throughout the year: autumn (October-23-2020), winter (February-12-2021), and spring (May-5-2021). Flowering shoots and inflorescences from nine FT7 plants were marked with ribbons and characterized at these time points. A total of 24 flowering shoots, approximately 3 shoots per plant, were labelled and monitored. To describe the flowering habit, several variables were analysed on each flowering shoot: (i) average length of shoots, (ii) percentage of shoots developing lateral shoots, (iii) average number of lateral shoots developed, (iv) average length of the lateral shoots, and (v) average number of inflorescences formed in each flowering shoot. Additionally, the following variables were recorded in each inflorescence: i) number of groups of flowers forming the inflorescence and ii) total number of flowers per inflorescence. These variables were recorded in, at least, 21 inflorescences.

### 2.8 Statistical analysis

Data were subjected to ANOVA, and mean separation was performed with the HSD-Tukey test using R. For non-parametric data a Kruskal-Wallis Rank Sum test was performed. Percentage data were analysed by Chi-squared test. All tests were performed at P=0.05.

## 3 Results

### 3.1 Phenotypical characterisation of *MtFTa1* olive plants in the confined greenhouse

**Figure 1:**
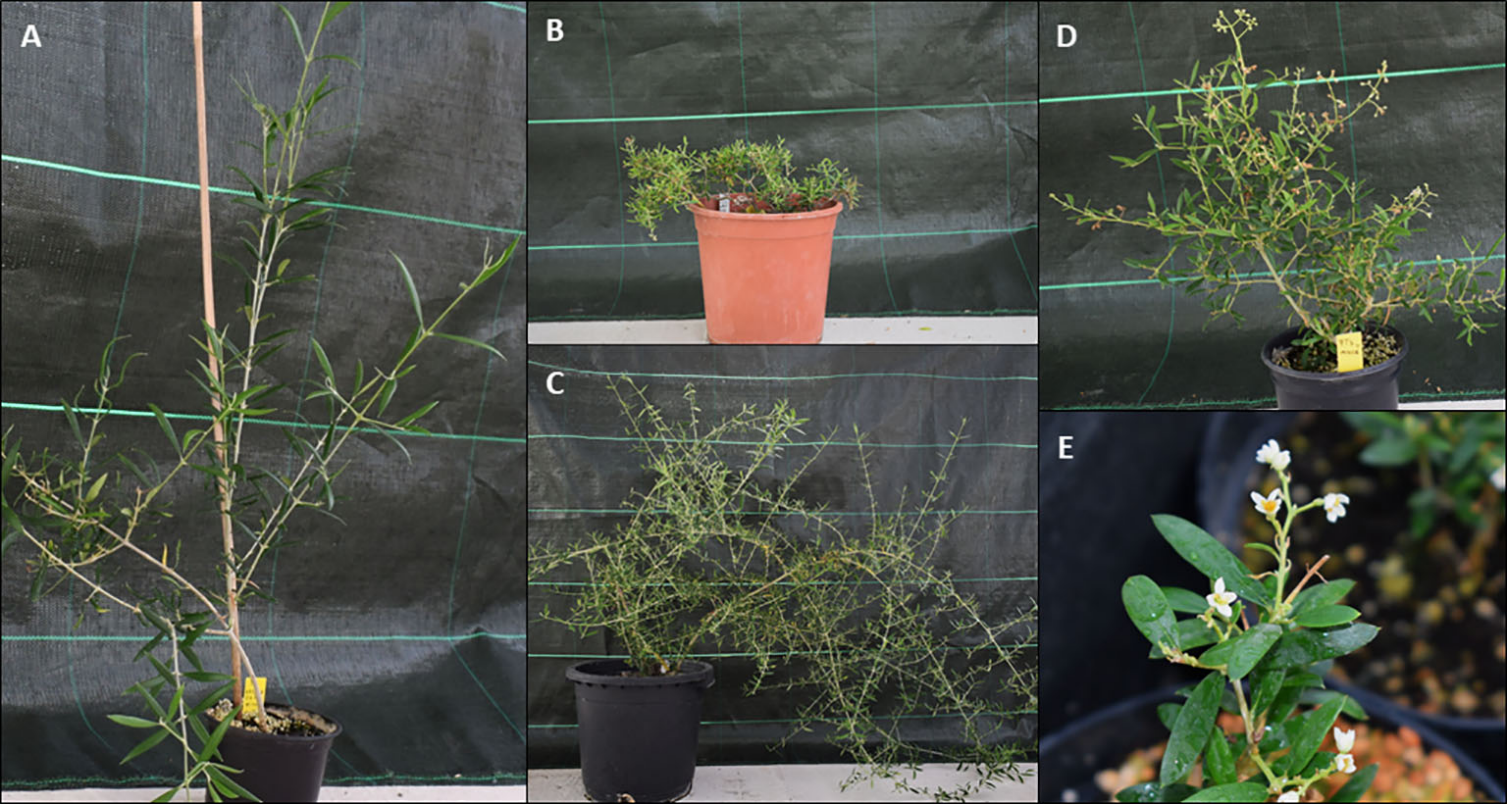
Control plant (P1) **(A)**, and 10-year-old olive transgenic plants transformed with *MtFTa1* gene from *Medicago truncatula*: FT15 **(B)**, FT5 **(C)** and FT7 **(D)** with a detail of its flowers **(E)**.

Haberman et al. (2017) obtained three olive transgenic lines (FT5, FT7 and FT15) transformed with the *MtFTa1* gene from *Medicago truncatula* under the control of the constitutive promoter CaMV35S; these lines flowered under *in vitro* conditions Micropropagation of the flowering lines was difficult since most vegetative buds gave rise to flower buds. Plants of the control and transgenic lines were acclimated in a growth chamber and some of the FT7 lines could be transferred to a confined greenhouse where they showed continuous flowering. The growth pattern and flowering behaviour of the three transgenic lines have been characterized in the present research. In successive years of growth in the confined greenhouse, control plants maintained the typical monopodial branching in olive, and no significant alterations in the growth habit were detected (**Figure 1A**). These plants were recurrently pruned to limit their growth. This treatment maintained control plants in a juvenile state, and no flowering was observed during the culture period.

Regarding the transgenic lines, plants from FT7 maintained the continuous flowering behaviour throughout successive years, while, by contrast, FT5 never developed flowers under greenhouse conditions (**Figures 1B–E**). FT7 plants also produced occasionally some fruits in the greenhouse. Flowering behaviour from FT15 plants was abnormal (only flower bud-like structures were observed, which did not show further development). Additionally, the three transgenic lines displayed a dwarf branching phenotype. The sympodial growth habit is not usual in olives, generally characterised by a solid apical dominance. In the case of FT7 and FT15, the conversion of apical meristems to floral buds led to the inhibition of apical meristem growth and the development of lateral shoots, resulting in dwarf, highly branched plants. Even though FT5 plants never flowered, the dwarf branching phenotype was also noticeable in this line.

### 3.2 Molecular characterisation of *MtFTa1* olive plants

**Figure 2:**
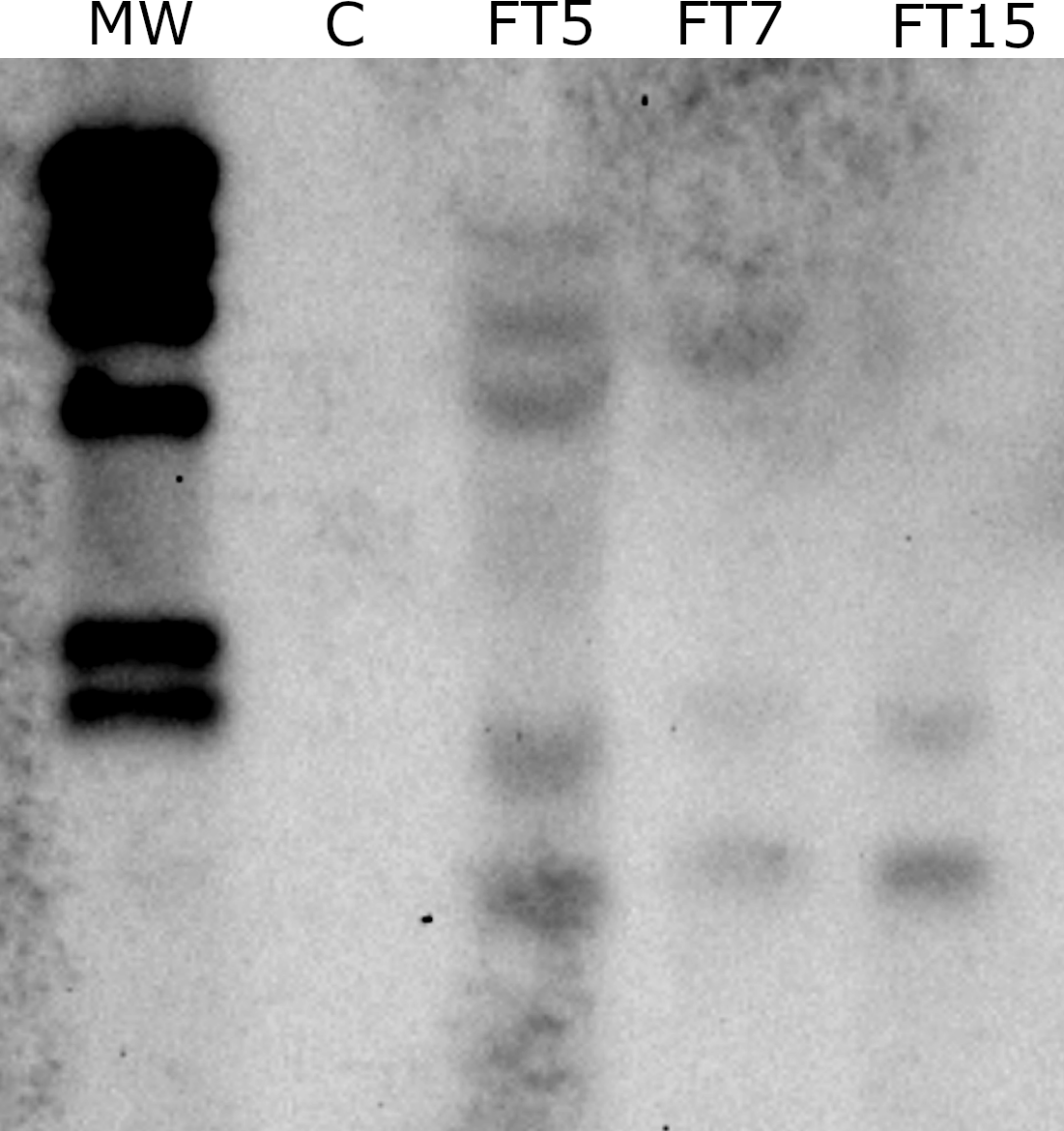
*Southern* blot analysis of *MtFTa1* gene in genomic DNA extracted from stem tips of control (P1) and transgenic olive plants (FT5, FT7 and FT15). Genomic DNA was digested with *XhoI* and *KpnI*, two enzymes that do not cut within the T-DNA of *35S:MtFTa1* plasmid. A 340 bp PCR product amplified from *MtFTa1*, labelled with DIG-dUTP was used as a probe. A digoxigenin-labelled λHindIII DNA (MW) is shown in lane 1.

**Figure 3:**
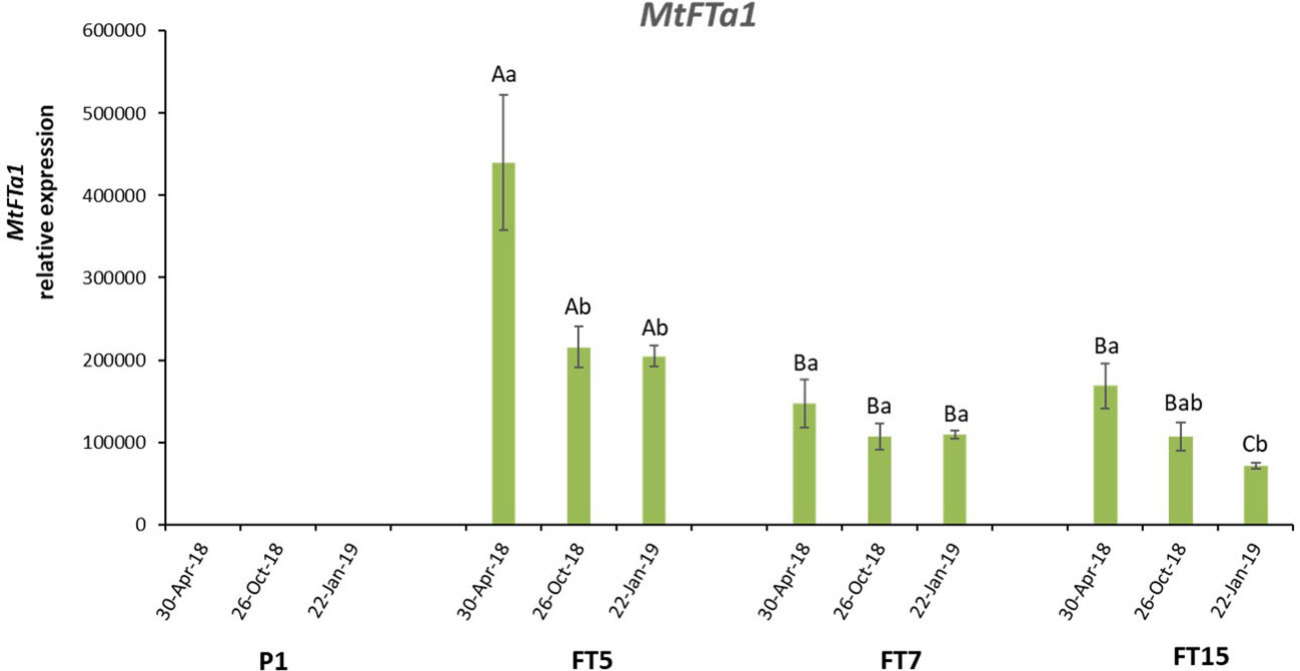
*MtFTa1* gene expression in olive transgenic plants, FT5, FT7 and FT15, and in control (P1) plants, along the year. Relative expression values were referred to control non-transformed plants, with the lowest expression level. Expression was measured in stem tips using *O. europaea* ubiquitin gene as reference. Each data point corresponds to the average of three independent biological repeats ± SE. Different lowercase letters over the bars indicate significant differences in the expression of every line throughout time, while uppercase letters show significant differences in the expression of the transgene among the different lines in every time point, according to the Tukey HSD test at P=0.05.

Olive *OeFT1* and *OeFT2* are 90% identical at mRNAs sequence level and their predicted proteins share around 84% of identity. Both genes show a 70% identity at mRNA level and 70% identity at protein sequence with the *Medicago* transgene *MtFTa1* (**Supplementary Figure 1**). The copy number of *MtFTa1* in the transgenic lines was determined by *Southern* blot using genomic DNA isolated from leaves. FT5, FT7, and FT15 lines contained 5, 3 and 2 copies of the transgene, respectively (**Figure 2**). As expected, no hybridisation signal was observed in control plants. Due to the differences in the phenotype found among the three lines, we decided to study the expression of the transgene by qRT-PCR in stem tips from the different lines along the year: spring (April-30-2018), when anthesis naturally occurs in field grown adult olives in Spain, autumn (October-26-2018) and winter (January-22-2019), towards the end of flower induction in field conditions (Haberman et al., 2017). The three transgenic lines overexpressed the *MtFTa1* gene throughout the year, with higher levels of transcripts in spring in the FT5 line (**Figure 3**). The highest levels of *MtFTa1* mRNA in all periods were detected in FT5 plants, the line with the highest copy number, also showing a pronounced dwarfing phenotype, but that never flowered under greenhouse conditions.

### 3.3 Expression of the flowering-related genes *OeFT1*, *OeFT2* and *OeTFL1-1* in transgenic plants

**Figure 4:**
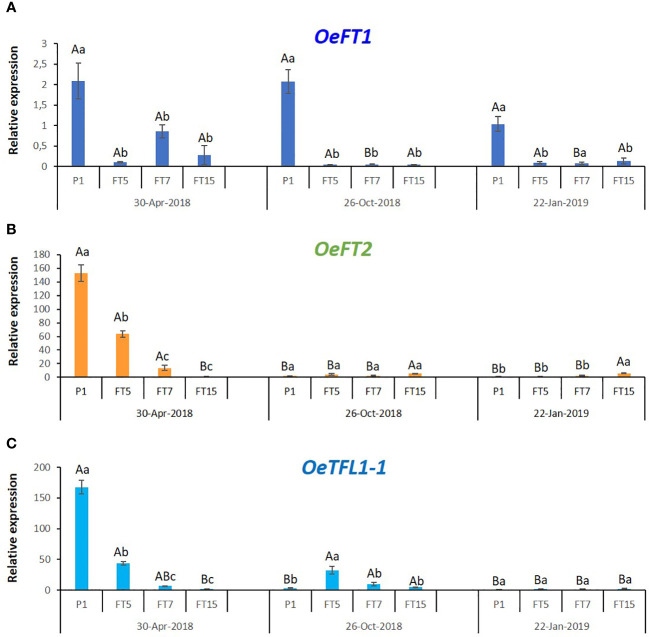
Expression of the endogenous olive genes *OeFT1* **(A)**, *OeFT2* **(B)**, and *OeTFL1-1* **(C)**, in control (P1), and transgenic FT lines throughout the year. Each data point corresponds to the average of three independent biological repeats ± SE. Relative expression values of every gene were referred to the control with the lowest expression level among the three periods (CP1, January-22-2019). Expression was measured in stem tips using *O. europaea* ubiquitin as reference gene. In every single graph, different lowercase letters over the bars represent significant differences in gene expression among the different lines in every time point, while different uppercase letters show significant differences in gene expression in every line throughout time, according to the Tukey-Kramer HSD test at P=0.05.

Previous research studied expression of FT-encoding olive genes in mature leaves, following expression in the same leaves, at different times from summer till the end of winter in trees exposed to outside conditions (Haberman et al., 2017). *OeTFL1-1* gene expression was detected in lateral meristems (Haberman et al., 2017). Here we were interested in studying the tissues that went through a change in growth pattern in the transgenic plants, the tips of shoots containing apical and lateral buds and the first two pairs of young developing leaf primordia. We followed the expression of the endogenous *FT* genes (*OeFT1* and *OeFT2*) as well as *OeTFL1-1* in these tissues, in control and transgenic plants grown in greenhouse conditions, at three timepoints during the year (**Figure 4**). In control, non-flowering plants there were no significant seasonal changes in *OeFT1* expression, while *OeFT2* and *OeTFL1-1* transcripts were significantly higher in spring. For the FT5 line, there were no significant seasonal changes in *OeFT1* expression, while *OeFT2* transcripts were significantly higher in spring. *OeTFL1-1* transcripts were significantly higher in spring and in fall. For the FT7 line, there were significantly higher levels in spring for both *OeFT1* and *OeFT2* transcripts. For the FT15 line, there were significantly lower levels in spring for *OeFT2* transcripts, and significantly higher levels of *OeTFL1-1* transcripts in the fall. The analysis of the expression of the endogenous flowering genes in the transgenic lines revealed that all of them were significantly under-expressed with respect to the control line in spring (**Figure 4**). The gene with expression in shoot tips showing correlation to flowering was *OeTFL1-1* in spring, since its expression was significantly higher in lines that did not flower: the control line and the FT5 line.

### 3.4 Endogenous contents of different plant hormones in *MtFTa1* olive transgenic plants

**Figure 5:**
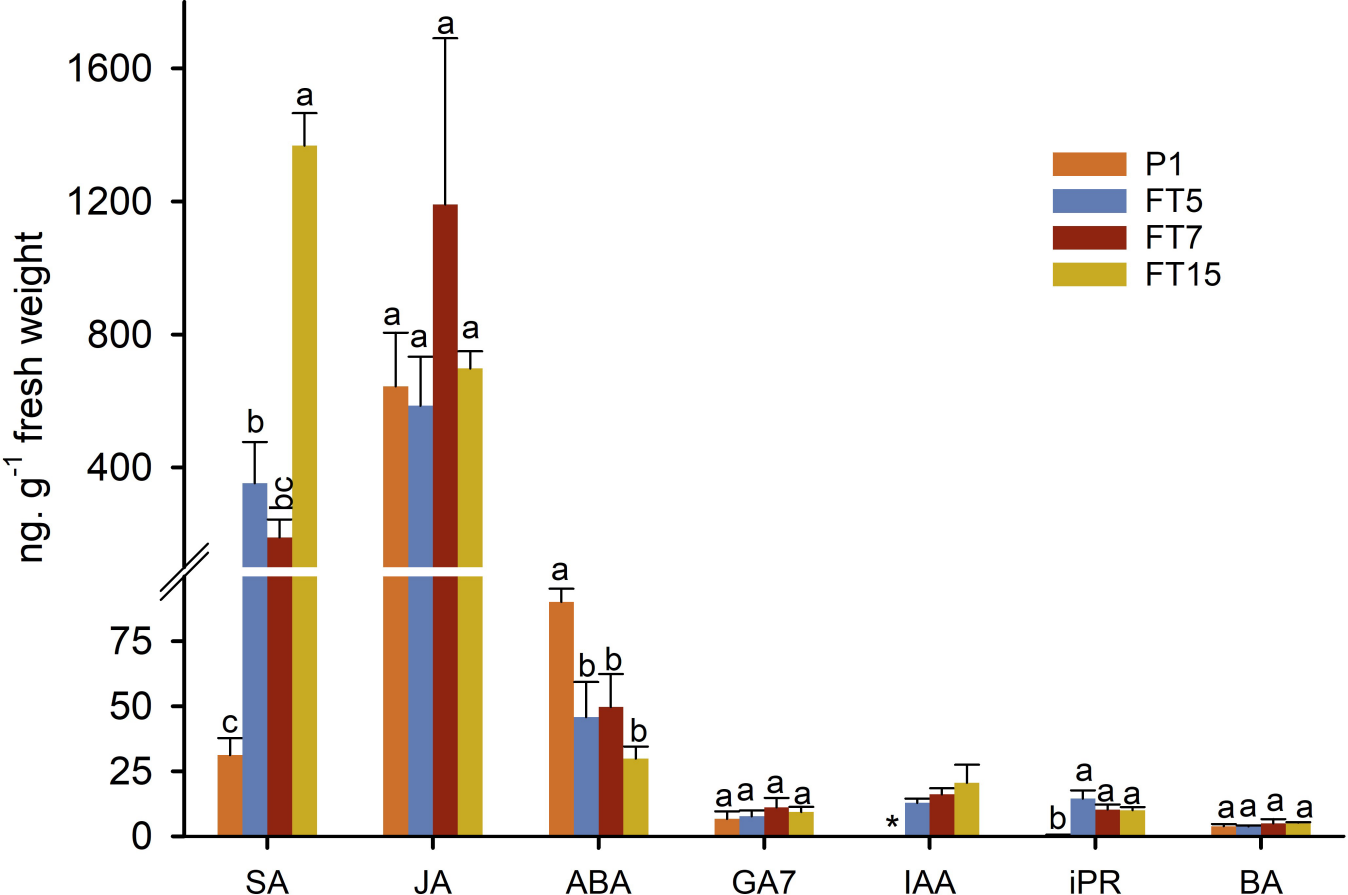
Hormonal content in stem tips collected in spring from control (P1) and transgenic olive plants expressing *MtFTa1* gene. Bars represent mean ± SD. For each hormone, bars with different letters indicate significant differences by Tukey test at P=0.05. SA, salicylic acid; JA, jasmonic acid; ABA, abscisic acid; GA7, gibberellin GA7; IAA, indol-3-acetic acid; iPR, isopentenyl adenosine; BA, benzyladenine. *IAA levels were too low and could not be accurately quantified in control samples.

We then asked whether hormone levels within the shoot tips correlated with flowering (not all transgenics) or with the growth phenotype (all transgenics). We analysed hormone levels of shoot tips in spring, since this was the season that showed interesting differences in gene expression (**Figure 4**). Apparently, no changes in any hormone level correlated with flowering (**Figure 5**). The unique branching behaviour of all the transgenic plants was correlated with significantly higher levels of salicylic acid (SA), indole acetic acid (IAA) and isopentenyl adenosine (iPR) and with significantly lower levels of ABA. We did not detect significant differences between the transgenic and non-transgenic lines for the hormones jasmonic acid (JA), GA7 and Benzyladenine (BA). No significant differences were observed within the three transgenic FT lines, except for FT15, which showed a significantly higher amount of SA than the other transgenic lines.

### 3.5 Characterisation of flowering in FT7 olive transgenic plants

The growth and flowering behaviour in FT7 transgenic plants, the line which exhibited continuous flowering, was characterised along the year (**Table 1**). The average length of the flowering shoots was similar on the three sampling dates. However, shoots developed more lateral shoots during spring. Furthermore, all lateral shoots were in flowering at this sampling date, while the percentage of lateral shoots in flowering decreased in autumn and winter. The length of the lateral shoots was also slightly higher in spring.

**Table 1:** Flowering related variables measured in FT7 olive transgenic plants.

	Spring	Autumn	Winter
Length of flowering shoots (cm)	13.6 ± 4.35^a^	15.3 ± 5.0 ^a^	14.3 ± 4.0 ^a^
Flowering shoots developing lateral shoots (%)	100^a^	37.5^c^	62.5^b^
Number of lateral shoots developed	4.9 ± 2.8 ^a^	2.4 ± 1.7^b^	2.9 ± 1.9 ^b^
Length of lateral shoots developed (cm)	3.3 ± 1.6 ^a^	2.8 ± 1.2 ^a^	2.8 ± 1.7 ^a^
Number of inflorescences per flowering shoot	5.2 ± 2.9 ^a^	1.8 ± 1.5 ^b^	1.9 ± 1.6 ^b^
Number of groups of flowers per inflorescence	5 ± 2 ^a^	7 ± 3 ^a^	3 ± 2 ^b^
Total number of flowers per inflorescence	16 ± 8 ^a^	19 ± 7 ^a^	12 ± 12 ^b^

Characterization of tagged flowering shoots measured at three time points along the year, in autumn (Oct-23-2020), winter (Feb-12-2021) and spring (May-05-2021). Numbers are mean values of 24 flowering shoots, from 9 different plants, ± SE (standard error). Regarding inflorescence characterization, each value corresponds to the mean value of 21 inflorescences ± SE. Different letters indicate significant differences in each variable over time, according to the Tukey-Kramer HSD test or to the non-parametric Kruskal-Wallis Rank Sum test, both at P=0.05. Percentages of flowering shoots developing lateral shoots were analysed by Chi-squared test at P=0.05.

There was an increase in the number of inflorescences per flowering shoot observed in spring with respect to autumn and winter. These inflorescences were formed by a variable number of groups of flowers, each one containing three flowers. The number of groups per inflorescence and, therefore, the total number of flowers per inflorescence were lower during winter (**Table 1**). A very small number of fruits was obtained during the year of analysis.

## 4 Discussion

The main goal of this work was to characterise olive plants transformed with the *MtFTa1* gene from *Medicago truncatula* to shed light on the unique phenotypes obtained by this transgene. For this purpose, three independent transgenic lines displaying some differences in the flowering and growth habits were studied along the year: autumn, winter, and spring. In other species, tomato (Lifschitz et al., 2014), pepper (Elitzur et al., 2009) or cotton (McGarry et al., 2016), an appropriate balance between TFL1-orthologues, flowering repressors, and FT-orthologues, flowering inductors, has been revealed to be essential in flower induction as well as in the determination of growth habit. For this reason, we studied the expression of these genes, as well as hormone levels in the different lines.

### 4.1 Control olive plants did not form flowers under greenhouse conditions probably because of an altered OeFT/OeTFL1-1 ratio

In this research, control and transgenic plants derived from the same embryogenic line were cultured for several years in a greenhouse with a cooling system, 30°C maximum temperature, average temperature during the winter season was 16°C, with a minimum temperature of 4.5°C. These conditions should be sufficient for winter flower induction in olive (Engelen et al., 2023). Non-transgenic plants of the same line flowered after 3-4 years when cultured under standard conditions (Bradaï et al., 2016). Here, our non-transgenic control plants had to be repeatedly pruned to control their growth in the greenhouse (maintenance of unpruned plants under confined conditions results in severe attacks of sooty mould and olive scale, probably due to lack of aeration). As a result, they were kept in a juvenile phase (did not flower) for nearly 10 years. Previous research showed that juvenile olive seedlings, compared to mature trees, expressed low levels of *OeFT2* in mature leaves towards the end of winter, and expressed higher levels of flower inhibitors (*OeTFL1* and *OeAP2*) in lateral meristems (Wechsler et al., 2022). In citrus, *CsTFL* transcript accumulation is associated with maintenance of juvenile traits (Pillitteri et al., 2004) while in *Arabis alpina*, *AaTFL1* expression prevents flowering in young plants and increase duration of cold treatments in older material (Wang et al., 2011). Here we studied expression in the shoot tip of the control and transgenic plants and noticed high levels of *OeTFL1-1* in the control plants in spring. This could explain the lack of flowering in the control plants.

High levels of *OeFT2* expression during spring were detected in shoot tips of control trees as well as FT5 and FT7 trees. Expression was higher in trees that did not flower (control and FT5). Does the OeFT2 protein have an additional function in shoot tips during spring? Besides inflorescence initiation, *OeFT2* could be responsible for the induction/maintenance of vegetative growth observed in spring. A role for an FT protein in shoot growth was documented in *Populus* (Brunner et al., 2014). The FT7 line, displaying continuous flowering, exhibited high levels of *OeFT1* mRNA in spring. The analysis of flowering-related parameters along the year showed that, although these plants formed flowers all year round, the flowering rate rose sharply in spring. In addition, the percentage of flowering branches that developed lateral shoots increased dramatically in spring (up to 100%) as well as the number of lateral shoots generated per flowering branch. Interestingly, MtFTa1 share 70% identity at protein level with OeFT1 and OeFT2, therefore, a negative effect of high MtFTa1 levels on the expression of endogenous FTs cannot be discarded.

### 4.2 Why does not the FT5 plant flower in the greenhouse?

Despite the very high levels of the *MtFTa1* transcript found in stem tips it is striking that FT5, the line with the highest transgene expression in all the periods, never flowered out of *in vitro* conditions. The fact that it did flower *in vitro*, and then stopped, might suggest that some culture conditions caused an epigenetic change in this line, that lead to repression of flowering in a plant with very high levels of FT. In *Arabidopsis*, hypomethylation of the FWA gene, which is normally only expressed in embryos, causes ectopic expression of the FWA protein in meristems. When expressed in meristems, this protein inhibits *Arabidopsis* flowering even in the presence of high FT (Soppe et al., 2000; Ikeda et al., 2007). Further studies should compare *MtFTa1* expression in mature leaves of these different transgenics as well as an FWA homolog in shoot tips. We did observe high levels of *OeTFL1-1* in shoot tips of this transgenic line during spring and fall, which might contribute to the lack of flowering.

### 4.3 The branching habit of the transgenic lines

Although the FT5 plant stopped flowering, it was highly branched. This trait was also observed in FT15 and FT7 plants. It could be attributed to one of the other functions of the FT protein in determination of plant architecture. The development of terminal flowers induced by the overexpression of *MtFTa1* interferes with the correlative inhibition effect of the apical meristem allowing the continuous growth of lateral shoots, as result, a highly branched, sympodial growth habit, unusual in olive, is observed. This branching habit has also been reported in *Eucalyptus* plants overexpressing the *AtFT* gene (Klocko et al., 2016) or in plum transformed with the *Populus trichocarpa* orthologue *PtFT1* gene, which not only showed continuous flowering but also exhibited alterations in their dormancy requirements; in addition, these plants exhibited shrub-type habit and panicle flowering architecture (Srinivasan et al., 2012).

### 4.4 Changes in hormone levels in the transgenic lines

Hormonal content in stem tips was measured in spring when control plants were actively growing and transgenic *MtFTA1* lines displayed an enhanced flowering phenotype. The main differences between control and transgenic plants were the reduced levels of ABA and the increments on iPR, IAA, and especially SA in FT lines. The three transgenic lines behaved similarly, except FT15, which showed increased SA compared to the other transgenic lines. Since the changes in hormone levels were common to all three transgenic lines, including FT5 that did not flower, one simple explanation is that these changes in hormone levels are associated with the unique plant architecture of these lines, and not to their flowering response. Still, if the flowering response in FT5 is blocked at an advanced stage of inflorescence differentiation, some of the changes in hormone levels might inform us on hormonal changes caused by FT, in the events leading to flowering. Thus, we will discuss possible roles for these hormones in flowering and plant architecture.

Salicylic acid induces the defence mechanism known as systemic acquired resistance (SAR), but this plant hormone can also induce flowering in some species (Cleland and Ajami, 1974; Martinez et al., 2004; Endo et al., 2009; Shah et al., 2021). In this research, the significantly higher levels of SA in transgenic plants suggest that FT can lead to an increase in SA levels with or without (FT5) leading to flowering. On the other hand, the enhanced lateral branched and reduced shoot length phenotype observed in transgenic olive plants resembled the phenotype of mutants or transgenic plants overproducing cytokinins (CKs) (Srivastava, 2002). The levels of iPR were significantly higher in stem tips from transgenic plants than in control. This fact could also explain the difficulty of rooting transgenic shoots *in vitro*, especially those from the FT5 line (data not shown), since an excess of CKs inhibits root formation. The role of CKs in regulating floral transition can differ between species. High cytokinin levels induced early flowering in *Arabidopsis* while cytokinin-deficient mutants exhibited a late-flowering phenotype (Izawa, 2021). However, exogenous application of 6-benzylaminopurine (BAP) to maize and rice plants delayed flowering time and reduced the expression of the florigen gene *Heading date 3a* (Cho et al., 2022). If the enhanced CK content is directly linked to FT overexpression needs further research. Surprisingly, IAA content was also higher in transgenic stem tips. Usually, both hormones are in close homeostasis, i.e. increased levels of IAA reduce free CKs and vice versa (Srivastava, 2002). This regulatory control was lost as a result of FT overexpression.

The role of ABA on olive floral induction is unclear. Baktir et al. (2004) suggested that the ratio GA/ABA could play a key role in this process; vegetative bud formation would be favoured if ABA levels were lower than GA, while the contrary favoured flower bud formation. In that study, both hormones were detected at similar concentrations, and also similar to the GA content reported in the present work; however, ABA levels in control and transgenic FT stem tips were several times higher than those reported by Baktir et al. (2004). In any case, the three transgenic lines contained significantly lower ABA than the control, challenging the hypothesis suggested by Baktir et al. (2004); however, this lower ABA content could be associated with the branching phenotype as demonstrated by Yao and Finlayson (2015) in *Arabidopsis thaliana*.

## 5 Conclusions

**Figure 6:**
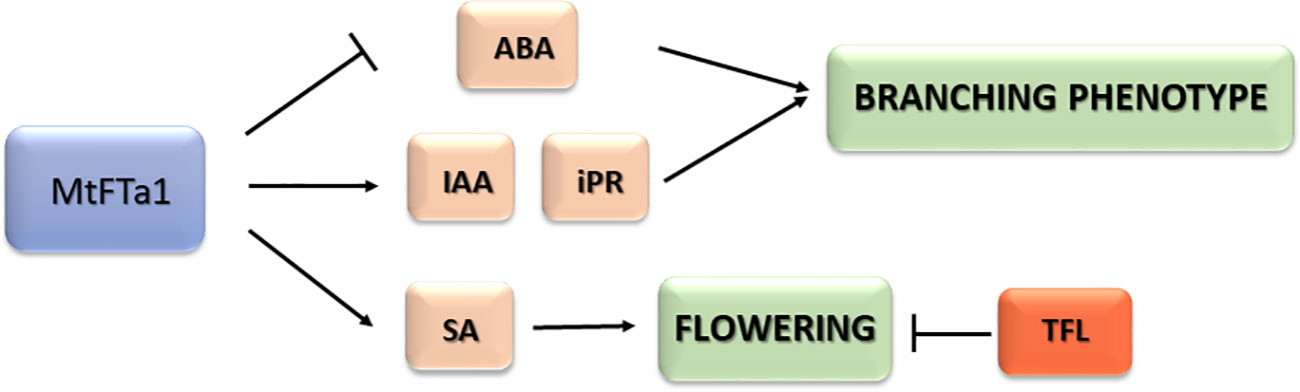
Summarizing scheme of hormonal changes and effects caused by MtFTa1 overexpression in transgenic olive plants.

Our results confirm that the *MtFTa1* gene from *Medicago* can function as effective florigen in a distant species such as olive, leading plants of some transgenic lines to flower continuously throughout the year, independently of environmental factors like temperature. However, flowering in the transgenic lines might also depend on low levels of *OeTFL1-1* in meristems. Interestingly, the heterologous expression of *MtFTa1* markedly modified the hormonal content of transgenic plants, highlighting the higher content of salicylic acid, IAA and isopentenyl adenosine (iPR) and lower values of ABA. The two main characteristics of the *MtFTa1* transgenic phenotype, continuous flowering and high branching, could be related to these hormonal changes (**Figure 6**). Further work can test whether reducing levels of one of these hormones, or inhibiting their activity, could affect one or both phenotypes. The development of novel olive varieties adapted to new environmental conditions is urgently needed. Because of climate change, warmer winters are expected, which can alter flowering time and reduce yield in traditional olive-growing areas. This work highlights the potential of a single transgene to modify vegetative and reproductive traits that could be very helpful in olive breeding and production.

## Data availability statement

The original contributions presented in the study are included in the article/**Supplementary Files**, further inquiries can be directed to the corresponding author.

## Author contributions

CG: Investigation, Writing – original draft. SC: Investigation, Writing – original draft. IF: Investigation, Writing – original draft. LR: Investigation, Writing – original draft. AS: Writing – review & editing. JM: Conceptualization, Funding acquisition, Methodology, Resources, Supervision, Writing – review & editing. FP-A: Conceptualization, Funding acquisition, Methodology, Project administration, Resources, Supervision, Writing – review & editing. EP-R: Conceptualization, Funding acquisition, Investigation, Methodology, Project administration, Resources, Supervision, Writing – original draft, Writing – review & editing.

## Supplementary material

Supplementary Figure 1 Multiple Alignment of FT proteins from *Medicago truncatula* (MtFTA1), Arabidopsis (AtFT) and Olive (OeFT1, OeFT2). Alignment was performed at the EMBL-EBI Website using the Clustal Omega tool (Sievers et al., 2011), with alignment presented using Jalview (Waterhouse et al., 2009). The colour of the fonts was based on Clustal.
