# Supplementary material for: Effect of heterologous expression of FT gene from Medicago truncatula in growth and flowering behavior of olive plants
Source: Front Plant Sci. 2024 Feb 22;15:1323087. doi: 10.3389/fpls.2024.1323087 (PMC10917891; doi:10.3389/fpls.2024.1323087)
Supplement: Supplementary Figure 1 — Multiple Alignment of FT proteins from Medicago truncatula (MtFTA1), Arabidopsis (AtFT) and Olive (OeFT1, OeFT2). Alignment was performed at the EMBL-EBI Website using the Clustal Omega tool (Sievers et al., 2011), with alignment presented using Jalview (Waterhouse et al., 2009). The colour of the fonts was based on Clustal. [file DataSheet_1.pdf]

```
MtFTa1 MAGSSRNPLAVGRVIGDVIDSFENSIPLRVTYGNRDVNNGCELKPSQIGNQPRVSVGGNDLRNLYTLVMVDPDSPSPSNPTFKEYLHWL
AtFT   MSINIRDPLIVSRVVGDVLDPFNRSITLKVTYGQREVTNGLDLRPSQVQNKPRVEIGGEDLRNFYTLVMVDPDVPSPSNPHLREYLHWL
OeFT2  -MSRDRDPLVVGRVIGDVLDPFIRSVSLEVTYGSREVNNGWDFRPSQILNPPRVDIGGDDLRTFYTLIMVDPDAPSPSDPNLREYLHWL
OeFT1  -MPRDRDPLVVGRIIGDVLDPFTRSVPLRIVYADREVNNGWDFRPSQIVNQPRVEIGGDDLRTFYTLIMVDPDAPSPSESSLREYLHWL

MtFTa1 VTDIPGTTEVTFGNEVVNYERPRPTSGIHRFVFVLFRQQCRQRVYAPGWRQNFNTREFAELYNLGSPVAAVFFNCQRESGSGGRTFR
AtFT   VTDIPATTGTTFGNEIVCYENPSPTAGIHRVVFILFRQLGRQTVYAPGWRQNFNTREFAEIYNLGLPVAAVFYNCQRESGCGGRRL-
OeFT2  VTDIPATTGASFGQEIVCYESPRPSMGIHRFVFALFRQLGRQTVYAPGWRQNFNTRDFAELYNLDLPVAAVYFNCQRESGTGGRRQ-
OeFT1  VTDIPATTGSSFGQEIVCYENPQPTMGIHRLVFVLFQQLGRQTVYAPGWRQNFNTREFAEIYNLGSPVAAVYFNCQRESGTGGRRS-
```
