# Supplementary material for: Effect of heterologous expression of FT gene from Medicago truncatula in growth and flowering behavior of olive plants
Source: Front Plant Sci. 2024 Feb 22;15:1323087. doi: 10.3389/fpls.2024.1323087 (PMC10917891; doi:10.3389/fpls.2024.1323087)
Supplement: Supplementary file 2 [file Table_1.docx]

***Table 1.*** List of primers used por probe synthesis in *MtFTa1* *Southern* analysis, and in Real Time PCR experiments.

| **Primer name** | **Sequence** | **Comments** |
| --- | --- | --- |
| **MtFTa1-For** | 5'-GGAAATCAACCGAGAGTGAG-3' | Used for probe synthesis in *MtFTa1* gene copy number studies in olive transgenic plants |
| **MtFTa1-Rev** | 5'-AAGAAGACAGCAGCAACAGG-3' |  |
| **MtFTa1-RTFor** | 5'-GCTGTAGGGCGTGTAATAGGG-3' | Used to detect relative expression of the transgene *(MtFTa1)* in olive transgenic plants |
| **MtFTa1RT-Rev** | 5'-TCACTCTCGGTTGATTCCAAT-3' |  |
| **OeFT1RTFor** | 5´-CTAGGGTTGAAATTGGTGGTGAT-3´ | Used to monitor *OeFT1* expression in olive transgenic plants |
| **OeFT1RTRev** | 5´-TGCAAGTATTCTCTGAGGCTAGAC-3´ |  |
| **OeFT2RTFor** | 5´-CCTTCGTACTTTCTACACGCTCATT-3´ | Used to monitor *OeFT2* expression in olive transgenic plants |
| **OeFT2RTRev** | 5´-TCAGTCACCAACCAGTGCAAA-3´ |  |
| **OeTFL1-1RTFor** | 5´-GGCAATAGACTAGTCTACAATGGACG-3´ | Used to monitor *OeTFL1-1* expression in olive transgenic plants |
| **OeTFL1-1RTRev** | 5´-GACATCAGGATCAATCATCACCAGT-3´ |  |
| **pUB1** | 5´-ATGCAGATCTTTGTGAAGAC-3´ | Used to amplify olive ubiquitin as reference gene in all the qRT-PCR experiments (Gómez-Jiménez *et al.*, 2010) |
| **pUB2** | 5´-ACCACCACGAAGACGGAG-3´ |  |
